# Supplementary material for: Magnetically tightened form-stable phase change materials with modular assembly and geometric conformality features
Source: Nat Commun. 2022 Mar 16;13:1397. doi: 10.1038/s41467-022-29090-1 (PMC8927617; doi:10.1038/s41467-022-29090-1)
Supplement: Supplementary file 1 — Supplementary Information [file 41467_2022_29090_MOESM1_ESM.pdf]

Supplementary Information

Magnetically tightened form-stable phase change materials with modular assembly and geometric conformality features

Yongyu Lu[1,2], Dehai Yu[1], Haoxuan Dong[1], Jinran Lv[1], Lichen Wang[2], He Zhou[3], Zhen Li[1], Jing Liu[2]*, Zhizhu He[1]*

[1] Department of Vehicle Engineering, College of Engineering, China Agricultural University, Beijing 100083, China.

[2] Key Laboratory of Cryogenics and Beijing Key Laboratory of Cyro-Biomedical Engineering, Technical Institute of Physics and Chemistry, Chinese Academy of Sciences, Beijing, 100190, China.

[3] School of Materials Science and Engineering, University of Science and Technology Beijing, Beijing, 100083, China.

*Email: jliu@mail.ipc.ac.cn and zzhe@cau.edu.cn.

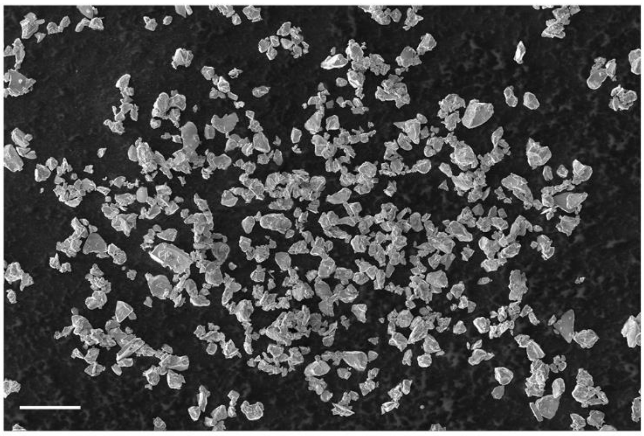

Supplementary Figure 1. Size distribution of NdFeB particles. Scale bar:50 μm.

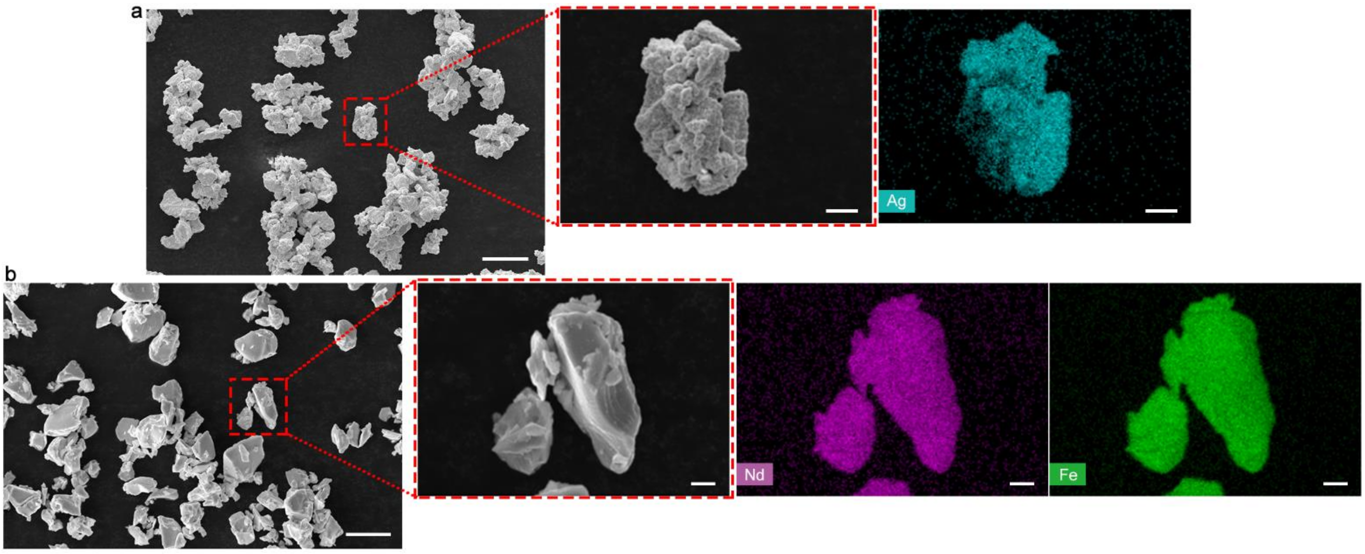

Supplementary Figure 2. SEM images and EDX element mappings of **a** NdFeB@Ag particles. Scale bars: 20 μm, 2 μm, 2 μm and **b** bare NdFeB. Scale bar: 10 μm, 1 μm, 1 μm, 1 μm.

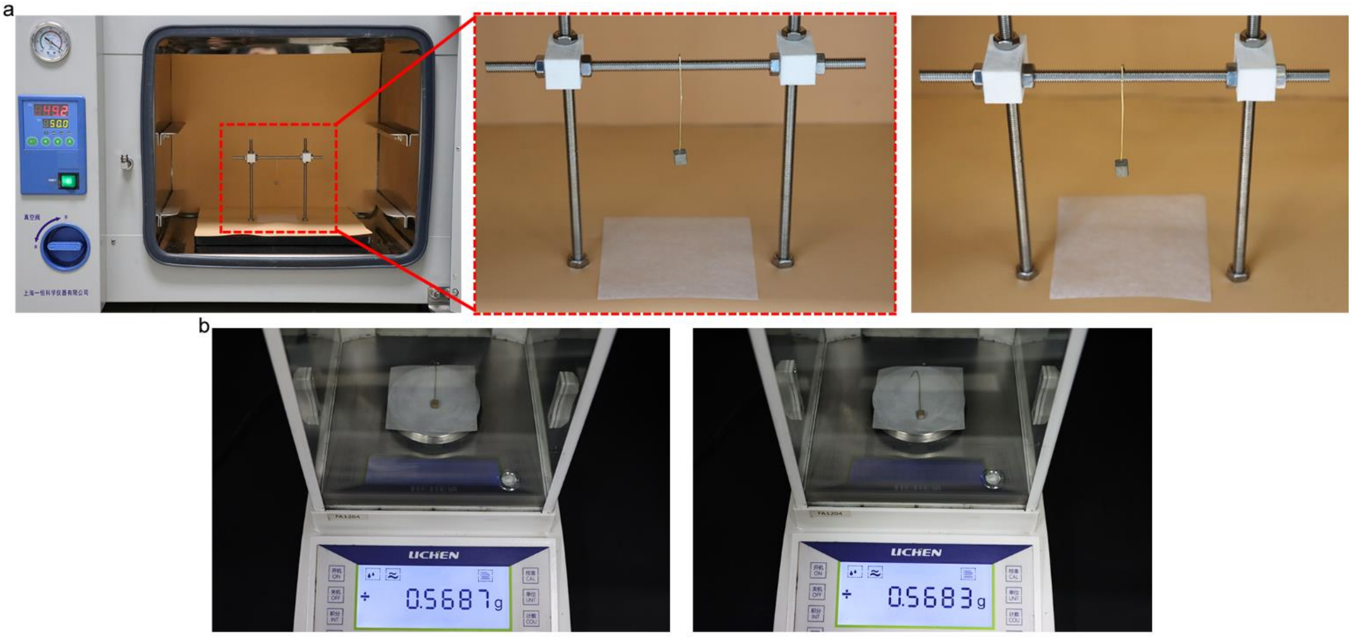

Supplementary Figure 3. Leakage-proof result of magnetically tightened form-stable phase change materials (MTPCMs). **a** Leakage test setup and the morphology of the MTPCM sample before and after heating. **b** The weight of the MTPCM sample before and after heating.

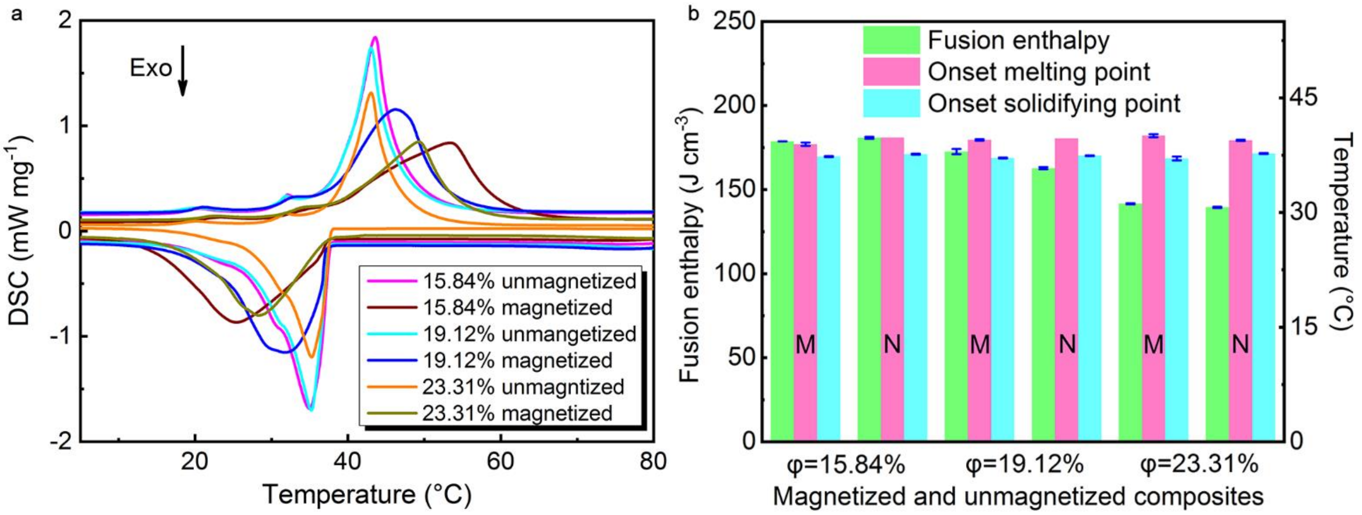

Supplementary Figure 4. Comparison of **a** DSC curves and **b** fusion enthalpy, onset melting, and solidifying point of magnetically tightened form-stable phase change materials (MTPCMs) and unmagnetized composites with the same volume ratio.

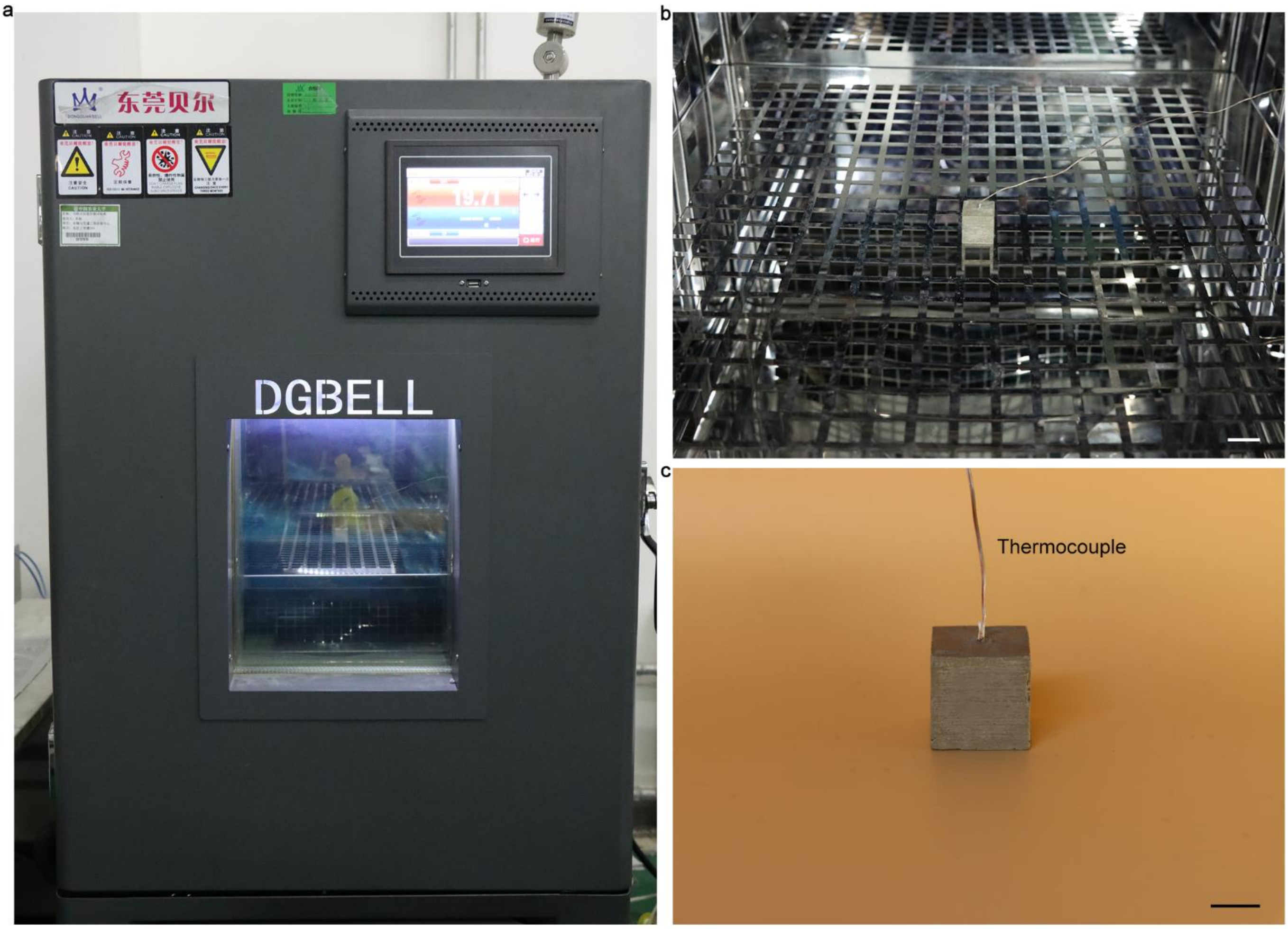

Supplementary Figure 5. a The incubator for heat charging/discharging test. Photographs of **b** the test

system and **c** the magnified sample. Scale bars: 20 mm, 10mm.

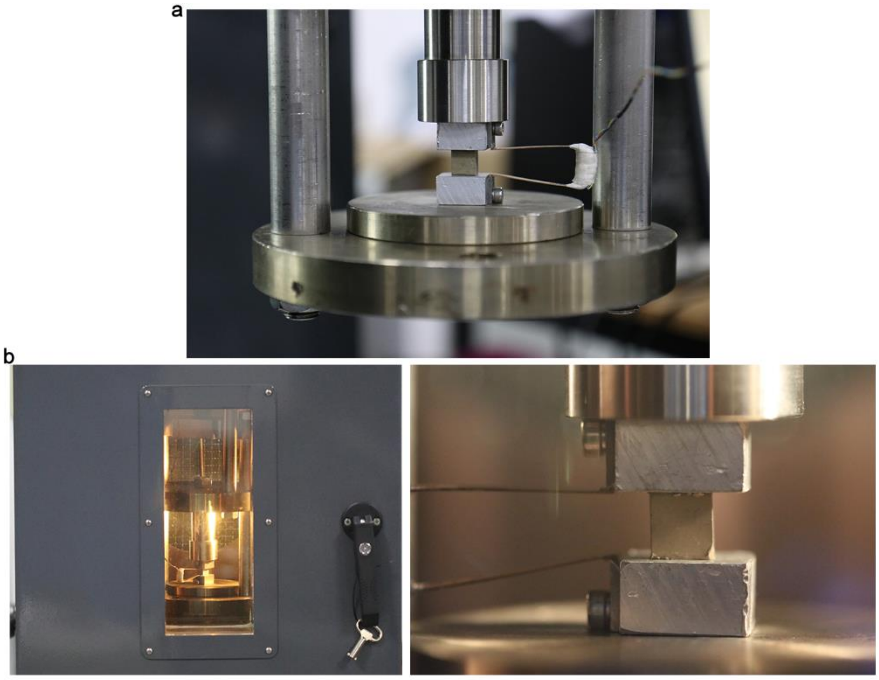

Supplementary Figure 6. Mechanical test setup for magnetically tightened form-stable phase change materials (MTPCMs). **a** The compression test system at room temperature. **b** The test system at the temperature above the melting point of paraffin.

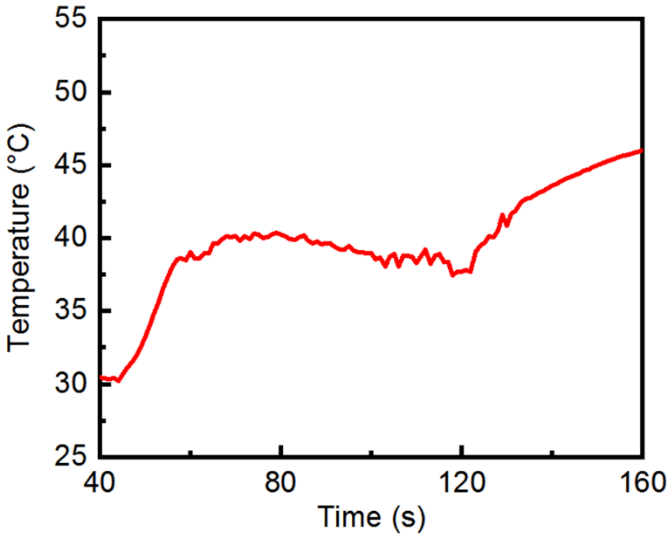

Supplementary Figure 7. Temperature variation of magnetically tightened form-stable phase change material (MTPCM) under a voltage of 3 V.

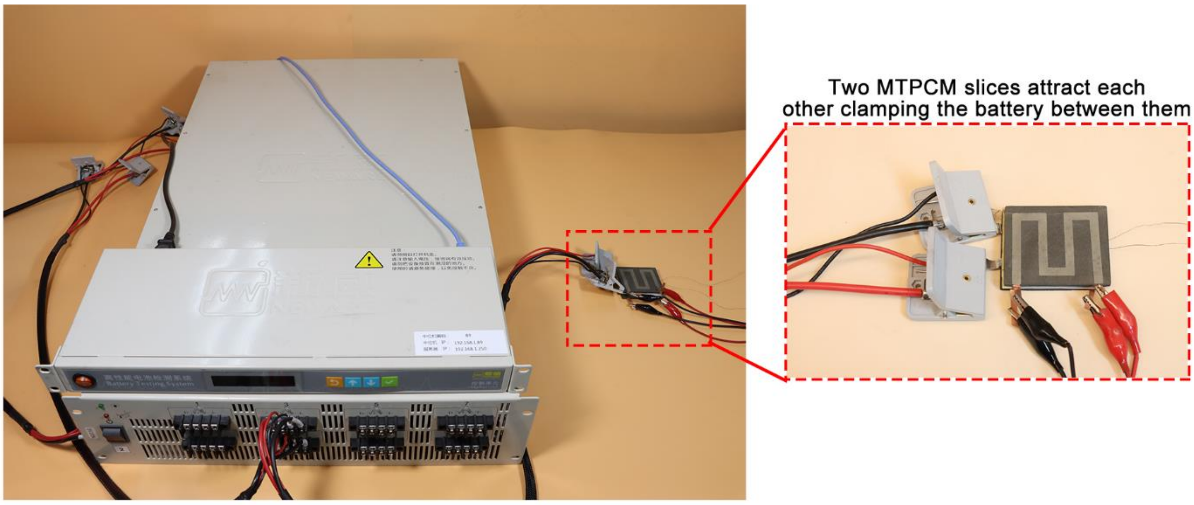

Supplementary Figure 8. Experiment setup of battery thermal management system.
