# Supplementary material for: Magnetically tightened form-stable phase change materials with modular assembly and geometric conformality features
Source: Nat Commun. 2022 Mar 16;13:1397. doi: 10.1038/s41467-022-29090-1 (PMC8927617; doi:10.1038/s41467-022-29090-1)
Supplement: Supplementary file 3 — Description of Additional Supplementary Files [file 41467_2022_29090_MOESM3_ESM.pdf]

Description of Additional Supplementary Files

File Name: Supplementary Movie 1
Description: Shape-stable and leakage-proof properties of magnetically tightened phase change materials during the phase change process.

File Name: Supplementary Movie 2
Description: Shape transformable and reconfigurable of magnetically tightened phase change materials.

File Name: Supplementary Movie 3
Description: Magnetically induced modular assembly of magnetically tightened phase change materials.

File Name: Supplementary Movie 4
Description: Compression test of magnetically tightened phase change materials at above the melting point.

File Name: Supplementary Movie 5
Description: Stretchable conductivity test of magnetically tightened phase change materials at above the melting point.

File Name: Supplementary Movie 6
Description: Heat-to electricity energy conversion of magnetically tightened phase change materials.

File Name: Supplementary Movie 7
Description: Temperature control capability of magnetically tightened phase change materials.
